# Resistance Monitoring of *Nilaparvata lugens* to Pymetrozine Based on Reproductive Behavior

**DOI:** 10.3390/insects14050428

**Published:** 2023-04-29

**Authors:** Xin-Yu Song, Yu-Xuan Peng, Yang Gao, Yan-Chao Zhang, Wen-Nan Ye, Pin-Xuan Lin, Cong-Fen Gao, Shun-Fan Wu

**Affiliations:** 1 College of Plant Protection, Nanjing Agricultural University, Nanjing 210095, China; 2 Sanya Institute of Nanjing Agricultural University, Sanya 572025, China; 3 State & Local Joint Engineering Research Center of Green Pesticide Invention and Application, Nanjing 210095, China

**Keywords:** *Nilaparvata lugens*, pymetrozine, reproductive behavior, bioassay method, resistance monitoring

## Abstract

**Simple Summary:**

Pymetrozine is one of the most common insecticides used to control the rice pest *Nilaparvata lugens* in China. Because of its unique mechanism of action and the fact that there is no obvious lethal effect after pymetrozine treatment, it is unreasonable to determine the sensitivity of pymetrozine by only calculating the mortality rate. In the context of traditional bioassay methods, which do not reflect pymetrozine’s control effect on the pest populations in the field, we established fecundity assay bioassay methods to monitor *N. lugens’* resistance level to pymetrozine, and systematically evaluated pymetrozine’s effect on the fecundity of *N. lugens*. Treatment with pymetrozine significantly reduced the number of offspring in both *N. lugens* nymphs and adults.

**Abstract:**

On the basis of the inhibition effects of pymetrozine on the reproductive behavior of *N. lugens,* we established a bioassay method to accurately evaluate the toxicity of pymetrozine in *N. lugens* and clarified the level of pymetrozine resistance of *N. lugens* in the field. In this study, pymetrozine’s effects on the fecundity of *N. lugens* were evaluated using the topical application method and rice-seedling-dipping method. Moreover, the resistance of *N. lugens* to pymetrozine in a pymetrozine-resistant strain (Pym-R) and two field populations (YZ21 and QS21) was determined using the rice-seedling-dipping method and fecundity assay methods. The results showed that treatment of *N. lugens* third-instar nymphs with LC_15_, LC_50_, and LC_85_ doses of pymetrozine resulted in a significantly reduced fecundity of *N. lugens*. In addition, *N. lugens* adults treated with pymetrozine, using the rice-seedling-dipping and topical application method, also exhibited a significantly inhibited fecundity. Using the rice-stem-dipping method, pymetrozine resistance levels were shown to be high in Pym-R (194.6-fold), YZ21 (205.9-fold), and QS21 (212.8-fold), with LC_50_ values of 522.520 mg/L (Pym-R), 552.962 mg/L (YZ21), and 571.315 (QS21) mg/L. However, when using the rice-seedling-dipping or topical application fecundity assay method, Pym-R (EC_50_: 14.370 mg/L, RR = 12.4-fold; ED_50_: 0.560 ng/adult, RR = 10.8-fold), YZ21 (EC_50_: 12.890 mg/L, RR = 11.2-fold; ED_50_: 0.280 ng/adult; RR = 5.4-fold), and QS21 (EC_50_: 13.700 mg/L, RR = 11.9-fold) exhibited moderate or low levels of resistance to pymetrozine. Our studies show that pymetrozine can significantly inhibit the fecundity of *N. lugens*. The fecundity assay results showed that *N. lugens* only developed low to moderate levels of resistance to pymetrozine, indicating that pymetrozine can still achieve effective control on the next generation of *N. lugens* populations.

## 1. Introduction

The brown planthopper (BPH), *Nilaparvata lugens* (Stål) (Hemiptera: Delphacidae), is a severe migratory rice pest in the Yangtze River Valley, in South and Southwest China [1,2]. There are three main ways in which the BPH damages rice plants. Firstly, the BPH ingests the rice sap through its mouthparts, which hinders the transport of nutrients and water in the rice plants. Secondly, females pierce the rice leaf sheaths with their spawning needles to dissipate plant water when spawning. Thirdly, the BPH transmits rice virus diseases such as rice grassy stunt virus and rice ragged shunt virus [3,4]. The BPH has a high innate capacity for proliferation, strong adaptability to the environment, and a long-distance migration ability, which makes it insidious, sudden, violent, and destructive. Therefore, the BPH presents a significant challenge for rice production in many Asian countries [5,6]. Chemical pesticides are still the main methods with which to control *N. lugens*. However, as a result of the long-term, widespread, and unscientific use of pesticides, *N. lugens* have developed high resistance levels to neonicotinoids, organophosphates, carbamates, and insect growth regulators [7,8,9]. Among them, imidacloprid, buprofezin, and thiamethoxam have been suspended for the control of *N. lugens* in China owing to their high resistance levels to them.

Pymetrozine is a pyridine azomethine insecticide with an excellent control effect against sucking pests [10]. It has been gradually recommended as one of the main insecticides for controlling BPHs in China since 2008 [9]. Pymetrozine can disrupt the normal function of the chordotonal mechanoreceptors, thereby affecting the insect’s sense of gravity, hearing, and coordination [11,12,13]. This, in turn, affects the feeding and reproductive behavior of the target insects. Our previous study demonstrated that pymetrozine inhibited the reproductive behavior of *N. lugens* by disrupting male initiative courtship, female abdominal vibration, and female oviposition. In addition, in *Drosophila*, a significant reduction in the male courtship index and female receptivity was observed after pymetrozine treatment [14]. Therefore, pymetrozine may significantly inhibit the reproductive behavior of *N. lugens* by interfering with hearing, thus effectively inhibiting the number of next-generation insects. However, the effects of pymetrozine’s systemic and contact activities on the fecundity of *N. lugens* have not been systematically studied.

In order to ensure the continuity of resistance monitoring and to take into account the actual operability of monitoring a large number of field populations, scientific research institutes in China have long been using the rice-stem-dipping method to monitor pymetrozine resistance in BPHs. This method evaluates the BPH’s toxicity to pymetrozine by introducing the third-instar nymphs onto rice stems treated with different pymetrozine concentrations and then assessing mortality after 7 days. The monitoring results showed that during 2012–2021, *N. lugens* field populations in China reached moderate to high resistance levels to pymetrozine, except for a few populations in 2012, which had sensitive to low resistance levels [9,15,16]. The LC_50_ values determined by the rice-stem-dipping method could only reflect the sensitivity of contemporary *N. lugens* to pymetrozine but could not reflect the pymetrozine inhibition in the next-generation *N. lugens* population. Hence, pymetrozine toxicity and *N. lugens* resistance could not be fully assessed.

Consequently, the Insecticide Resistance Action Committee (IRAC) and scientists from Japan have established bioassay methods to evaluate the activity of pymetrozine against *N. lugens* based on treating contemporary *N. lugens* with pymetrozine while investigating the number of next-generation nymphs [17]. The IRAC no.005 method (IRAC no.005) introduces adult BPH females and males onto potted rice plants dipped in different concentrations of pymetrozine solution. Then, they are removed after 7 days of spawning, and the number of offspring is counted after 18 days. This method can investigate pymetrozine’s effect on contemporary and next-generation test insects under both systemic and contact modes of action. However, this method demands a large space and has a long test cycle due to the preparation of potted rice plants. Tsujimoto et al. only examined the contact effect of pymetrozine. Different doses of pymetrozine were topically applied to female brown planthoppers using a micro-applicator, and the males were not treated. Thereafter, they were transferred to rice seedlings in a test tube to spawn for 7–8 days and then removed. After 15–16 days, the number of offspring was counted. This method can only detect pymetrozine’s effect on female receptivity and oviposition behavior, but cannot reflect the pymetrozine’s effect on male courtship, male fertility, or the systemic activity of pymetrozine. In summary, the above two methods were used for test insects that had mated for many days and each replication introduced multiple females and males. However, there are certain limitations to the study. Firstly, the age and time of death of each female in each replication could not be determined, thus making it impossible to ensure that each female spawned for the same duration. Secondly, it was not possible to accurately determine whether each female mated or not, and thus, it was impossible to accurately assess the effects of pymetrozine on courtship, receptivity, and oviposition in brown planthoppers. Therefore, there is still a relative lack of effective resistance monitoring technology to assess pymetrozine resistance in *N. lugens*. In addition, the real resistance level of field *N. lugens* populations to pymetrozine still needs to be established, and these data, when available, will significantly affect resistance monitoring and the management of pymetrozine in relation to *N. lugens*. Accordingly, in order to comprehensively evaluate the effect of pymetrozine on *N. lugens* mating and female reproduction, the two fecundity assay methods established in our research used newly emerged unmated *N. lugens* for single-pair pairing. In addition, only treatments with surviving females after 7 days were counted in order to exclude the effects of oviposition duration on the number of offspring and to improve the accuracy of the test results. The fecundity assay method can comprehensively reflect pymetrozine’s persistence, its characteristics in terms of inhibiting reproductive behavior, and more objectively reflect pymetrozine’s toxicity against BPHs.

In this study, we found that pymetrozine could significantly inhibit the reproductive behavior of *N. lugens*. Pymetrozine treatment can significantly inhibit the fecundity of *N. lugens* in nymphs and adults. On this basis, a more accurate bioassay method, i.e., the fecundity assay method, was established to reflect the pymetrozine resistance of *N. lugens*. In addition, this method was used to monitor pymetrozine resistance in *N. lugens* both in laboratory and field populations. These results are valuable for the monitoring and management of pymetrozine resistance.

## 2. Materials and Methods

### 2.1. Insects

The susceptible strain (Pym-S) of BPH was initially collected from Hangzhou, Zhejiang Province, in 1995 and has been maintained in the laboratory without exposure to any insecticides since. The pymetrozine-resistant strain (Pym-R) was collected from Jinhua, Zhejiang Province, in 2013 and was selected for resistance to pymetrozine for more than 90 generations. Two field populations (QS21 and YZ21) were collected from Qianshan (30°37′ N, 116°34′ E), Anhui Province, and Yizheng (32°16′ N, 119°11′ E), Jiangsu Province, in September 2021, respectively. All the populations were reared on indica rice seedlings (Taichung Native 1, TN1) under standard conditions of 27 ± 1 °C, 70–80% relative humidity, and a 16 h light/8 h dark photoperiod.

### 2.2. Insecticides

Technical-grade pymetrozine (95%) was supplied by Jiangsu Anpon Electrochemical Co., Ltd. (Huaian, China). The technical-grade pymetrozine was dissolved in acetone (for topical application) or *N*,*N*-dimethylformamide (for systemically application) as stock solution. Then, a serial dilution was prepared using acetone for the topical application bioassay and water containing 0.1% Triton X-100 for the rice-stem-dipping bioassay.

### 2.3. Bioassays

#### 2.3.1. Rice-Stem-Dipping Bioassay Method

The rice-stem-dipping bioassay method was used to evaluate the LC_15_, LC_50_, and LC_85_ estimations [18]. Briefly, pymetrozine solution was diluted 4–5 concentrations in equal proportion and 3–4 replicates for each concentration. Three rice stems were grouped together and dipped in pymetrozine solutions for 30 s and then air-dried at room temperature. The roots of rice stems were wrapped with water-impregnated cotton and put into plastic cups. Fifteen insects (third-instar nymphs) were introduced onto rice stems for each replicate. Control rice stems were treated with 0.1% Triton X-100 water solution only. The plastic cups with treated insects were maintained under standard conditions of 27 ± 1 °C and 70–80% relative humidity, with a 16 h light/8 h dark photoperiod. Mortality was assessed after 168 h of exposure to pymetrozine.

#### 2.3.2. Fecundity Assay Bioassay Method

The rice-seedling-dipping fecundity assay method was used to evaluate the EC_50_ estimations. The no.005 method was referred to and modified (IRAC no.005). Firstly, 30-day-old rice seedlings (approximate) were dipped in a series of concentrations of pymetrozine for 30 s each. Then, each newly emerged virgin female adult was paired with a single male onto the rice seedlings. After 7 days, the adults were removed, and their survival was counted. After 15 days, the number of nymphs (only counting the replicates of female adults surviving after 7 days) was counted, and the inhibition rate of offspring under the treatment of the corresponding pymetrozine concentration was calculated. There were 30 replicates for each concentration and 4–5 doses of pymetrozine. Control rice stems were treated with 0.1% Triton X-100 water solution only. All treatments were maintained under standard conditions of 27 ± 1 °C and 70–80% relative humidity, with a 16 h light/8 h dark photoperiod.

The topical application fecundity assay method was used to evaluate the ED_50_ estimations. The method by Katsuhiko Tsujimoto that combines the topical application and the determination of offspring number was referred to and modified [17]. Newly emerged virgin female adults were anesthetized with carbon dioxide for 10 s. A droplet (0.2 μL) of insecticide acetone solution was applied topically to the prothorax notum of each individual female with a hand-held micro-applicator (Hamilton Repeating Applicator, Burkard Manufacturing Co., Ltd., Rickmansworth, UK). Males were not treated, and control groups were treated with acetone. Then, each treated female adult was paired with a single male onto the 30-day-old rice seedlings (approximate). The remaining steps and experiment conditions were consistent with rice-seedling-dipping fecundity assay method.

The inhibition rate (%) was calculated using the following formula:

$$\mathrm{Inhibition}\;\mathrm{rate}\;(\%)=\frac{\begin{array}{c} Number\;of\;nymphs\;in\;the\;control\;group\;-\\ Number\;of\;nymphs\;in\;pymetrozine-treatment\;group \end{array}}{\mathrm{Number}\;\mathrm{of}\;\mathrm{nymphs}\;\mathrm{in}\;\mathrm{the}\;\mathrm{control}\;\mathrm{group}}\times 100$$

### 2.4. Effects of Pymetrozine Treatment at Third-Instar Nymph Stage on the Fecundity of N. lugens

The LC_15_, LC_50_, and LC_85_ concentrations of pymetrozine in the susceptible strain (Pym-S) of BPH was determined by rice-stem-dipping bioassay method. Additionally, then the third-instar nymphs were introduced onto rice seedlings treated with pymetrozine solutions at LC_15_, LC_50_, and LC_85_ concentrations. After 7 days, the nymphs were transferred to new rice seedlings without pymetrozine treatment and reared until they emerged as adults. The newly emerged unmated *N. lugens* adults were paired according to the following combinations: ♀t (treated female) × ♂ck (untreated male); ♀ck (untreated female) × ♂t (treated male); and ♀t (treated female) × ♂t (treated male). Single pairs were introduced onto 30-day-old TN-1 rice seedlings to spawn for 7 days, and the number of offspring was counted after 15 days. ♀ck (untreated female) × ♂ck (untreated male) were regarded as the control group. There were no less than 30 replicates for each treatment.

### 2.5. Effects of Pymetrozine Treatment at Adult Stage on the Fecundity of N. lugens

The rice-seedling-dipping treatment and the susceptible strain (Pym-S) were used to evaluate the systemic toxicity of pymetrozine in BPHs. To this end, 30-day-old TN-1 rice seedlings (approximate) were dipped in 10 mg/L pymetrozine solution for 30 s and then air-dried at room temperature. Control rice seedlings were treated with 0.1% Triton X-100 water solution only. Pymetrozine’s effect on the fecundity of *N. lugens* was studied according to two treatments. The first treatment involved introducing single pairs of newly emerged unmated female and male adults in order to investigate the effects of pymetrozine on mating and reproduction in *N. lugens*. The second treatment involved introducing single *N. lugens* females that had emerged 2 to 3 days previous and were fully mated in order to investigate the effects of pymetrozine on oviposition in BPHs. In both of the above treatments, we removed the adults after 7 days, and the number of offspring was counted after 15 days. There were no less than 30 replicates for each treatment.

The topical application treatment and the susceptible strain (Pym-S) were used to evaluate the contact toxicity. *N. lugens* adults were anesthetized with carbon dioxide for 10 s. A droplet (0.2 μL) of 0.1 mg/L pymetrozine solution (0.02 ng/adult) was topically applied to the prothorax notum of the following adults with a hand-held micro-applicator (Hamilton Repeating Applicator, Burkard Manufacturing Co., Ltd., Rickmansworth, UK): (1) unmated females; (2) unmated males; (3) unmated females and males; and (4) fully mated pregnant female adults. Corresponding control groups were treated with acetone only. The topically applied *N. lugens* was introduced onto 30-day-old TN-1 rice seedlings as follows: unmated females paired with untreated males (♀t × ♂ck); unmated males paired with untreated females (♀ck × ♂t); unmated females paired with unmated males (♀t × ♂t); mated pregnant females (♀t). We also removed the adults after 7 days, and the number of offspring was counted after 15 days. There were no less than 30 replicates for each treatment.

### 2.6. Data Analysis

The sub-lethal (LC_15_), median lethal (LC_50_) and highly lethal (LC_85_) concentrations and their 95% fiducial limits (FL) were calculated using the POLO-plus program (Version 2.0) (LeOra Software LLC, Berkeley, CA, USA) for BPHs. The median effective concentrations (EC_50_) and the median effective dose (ED_50_) were estimated using the GraphPad Prism 8 Software (GraphPad Software Inc., San Diego, CA, USA). Data statistical analyses were conducted using the GraphPad Prism 8.0 software (GraphPad Software Inc., San Diego, CA, USA). Data from multiple groups were assessed using one-way ANOVA with post hoc Tukey HSD, and the data between two groups fitted with normal distribution were assessed using Student’s *t*-test, and the Mann–Whitney test was used to evaluate non-parametric. The resistance ratio (RR) was calculated by dividing the LC_50_, EC_50_, or ED_50_ value of a resistant strain by that of the susceptible strain. Insecticide resistance of the field populations was classified as follows: RR < 5-fold as susceptible; RR = 5–10-fold as low resistance; RR = 10–100-fold as medium resistance; and RR > 100-fold as high resistance [9].

## 3. Results

### 3.1. Pymetrozine Toxicity Determination in N. lugens Third-Instar Nymphs Using the Rice-Stem-Dipping Method

Pymetrozine toxicity in the *N. lugens* third-instar nymphs was determined using the rice-stem-dipping method. The results showed that the LC_15_, LC_50_, and LC_85_ values of pymetrozine to the pymetrozine-susceptible strain (Pym-S) were 0.422 mg/L, 2.685 mg/L, and 17.073 mg/L, respectively (Table 1). The LC_50_ values of the pymetrozine-resistant strain (Pym-R) and two field populations (QS21 and YZ21) were 522.520 mg/L, 571.315 mg/L, and 552.962 mg/L, respectively. All three populations developed high levels of resistance to pymetrozine, with resistance ratios of 194.6-fold, 212.8-fold, and 205.9-fold, respectively (Table 1).

### 3.2. Pymetrozine Toxicity Determination in N. lugens Adults Using the Fecundity Assay Method

The fecundity assay method was developed based on the fact that pymetrozine can interfere with the reproductive behavior of *N. lugens* [14]. Pymetrozine’s toxicity in *N. lugens* was determined by counting the inhibition rate of different pymetrozine doses on the number of *N. lugens* offspring. As shown in Table 2, the EC_50_ and ED_50_ values for the pymetrozine-susceptible strain (Pym-S) were 1.155 mg/L and 0.052 ng/adult, respectively, which were used as the baseline to monitor the resistance of BPHs to pymetrozine using the fecundity assay method.

Inhibition of the fecundity of *N. lugens* as a result of the systemic activity of pymetrozine was determined using the rice-seedling-dipping fecundity assay method. The results indicated that Pym-R strain (EC_50_: 14.370 mg/L, RR = 12.4-fold), YZ21 (EC_50_: 12.890 mg/L, RR = 11.2-fold), and QS21 (EC_50_: 13.700 mg/L, RR = 11.9-fold) developed a moderate level of resistance to pymetrozine (Table 2). Inhibition of the fecundity of *N. lugens* resulting from the contact activity of pymetrozine was determined using the topical application fecundity assay method. The results indicated that Pym-R (ED_50_: 0.560 ng/adult, RR = 10.8-fold) and YZ21 (ED_50_: 0.280 ng/adult; RR = 5.4-fold) developed a low or moderate level of resistance to pymetrozine (Table 2). Our results indicate that both pymetrozine’s systemic toxicity and contact toxicity can effectively inhibit the offspring of *N. lugens*.

### 3.3. Effects of Pymetrozine Treatment at Third-Instar Nymph Stage on the Fecundity of N. lugens

**Figure 1:**
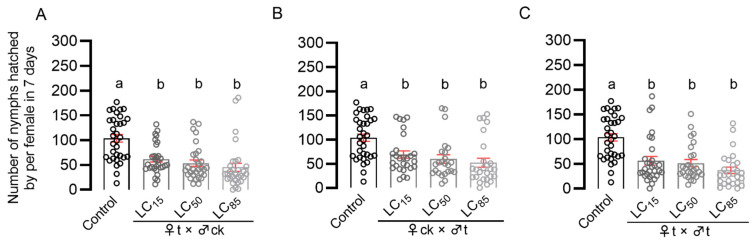
Effects of pymetrozine treatment at third-instar nymph stage on the fecundity of *N. lugens*. Single female fecundity after emergence of *N. lugens* nymphs treated with sub-lethal, median, and high concentrations of pymetrozine ((**A**) ♀t × ♂ck; (**B**) ♀ck × ♂t; and (**C**) ♀t × ♂t). The variance analysis of all data was performed by one-way ANOVA with post hoc Tukey HSD. Error bars represent SE. Different lowercase letters showed significant differences at the 0.05 level (*p* < 0.05).

In order to explore pymetrozine’s effect on the reproductive behavior of *N. lugens*, sub-lethal (LC_15_), median lethal (LC_50_), and highly lethal (LC_85_) concentrations of pymetrozine were used to treat third-instar pymetrozine-susceptible strain nymphs, as shown in Table 1. The number of offspring treated with three lethal concentrations is presented in Figure 1, as compared with the control group (♀ck × ♂ck), the number of offspring in the LC_15_, LC_50_, and LC_85_ pymetrozine-treated female (♀t × ♂ck) groups was reduced by 40.7 %, 48.4 %, and 56.4 %, respectively (Figure 1A). In addition, the number of offspring in the pymetrozine-treated male (♀ck × ♂t) group was significantly reduced by 33.5 %, 42.3 %, and 49.7 %, respectively (Figure 1B), for the aforementioned concentrations. Moreover, the number of offspring in the pymetrozine-treated female and male (♀t × ♂t) groups was significantly reduced by 45.6 %, 50.3 % and 64.3 %, respectively (Figure 1C), for the aforementioned concentrations. Therefore, pymetrozine treatment at the *N. lugens* third-instar nymph stage was shown to continuously inhibit male fertility and female fecundity and lead to a significant decrease in the number of offspring.

### 3.4. Effects of Pymetrozine Treatment at Adult Stage on the Fecundity of N. lugens Using the Rice-Seedling-Dipping Method

**Figure 2:**
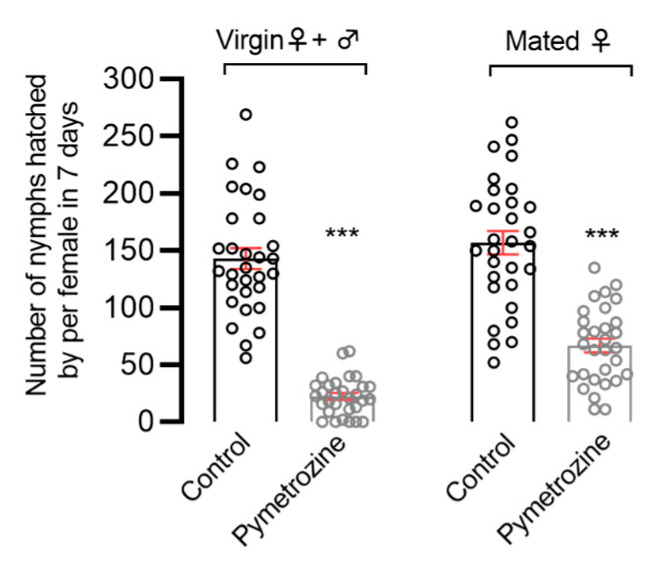
Effects of pymetrozine treatment on the fecundity of *N. lugens* adults using the rice-seedling-dipping method. The variance analysis of all data was performed using Student’s *t*-test. Error bars represent SE (*** *p* < 0.0001).

The effect of pymetrozine’s systemic activity on the fecundity of *N. lugens* was determined using the rice-seedling-dipping method. As shown in Figure 2, the number of offspring in pymetrozine-treated unmated females’ and males’ group was significantly reduced by 84.3%, and in pymetrozine-treated mated females’ group was significantly reduced by 57.3% as compared with the control group. These results indicate that pymetrozine can inhibit *N. lugens* fecundity via the systemic route.

### 3.5. Effects of Pymetrozine Treatment at Adult Stage on the Fecundity of N. lugens Using the Topical Application Method

**Figure 3:**
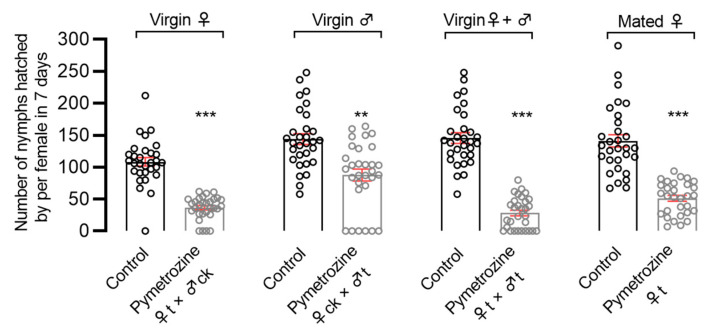
Effects of pymetrozine treatment at the adult stage on the fecundity of *N. lugens* using the topical application method. Statistical analysis of normal distribution data was performed using Student’s *t*-test and statistical analysis of abnormal distribution data was performed using Mann–Whitney test. Error bars represent SE (** *p* < 0.001; *** *p* < 0.0001).

The effect of pymetrozine’s contact activity on the fecundity of *N. lugens* was determined using the topical application method. The results showed that the number of offspring in the four topically applied combinations: unmated females paired with untreated males (♀t × ♂ck); unmated males paired with untreated females (♀ck × ♂t); unmated females paired with unmated males (♀t × ♂t); and mated pregnant females (♀t), was significantly reduced by 66.4%, 35.8%, 80.6%, and 63.7%, respectively, as compared with the control group (Figure 3). These results indicate that pymetrozine can inhibit the *N. lugens* fecundity via contact route.

Moreover, treating unmated adults with pymetrozine increased the number of no-offspring females whether using the topical application method or rice-seedling-dipping method. Above-mentioned results indicate that pymetrozine exhibits strong inhibitory effects on next-generation BPH populations.

## 4. Discussion

Pymetrozine has been recommended as an alternative insecticide to control BPH, which has developed resistance to imidacloprid, thiamethoxam, and buprofezin [9,19]. Using the rice-stem-dipping bioassay method, it was shown that BPHs have developed a strong resistance to pymetrozine [9,16]. However, pymetrozine still exhibits a good control effect against BPHs in paddy fields. Therefore, it is necessary to establish another bioassay method to evaluate the current resistance of BPHs to pymetrozine. On the basis of the impact of pymetrozine on BPHs reproductive behavior, we established a fecundity assay bioassay method and found that BPHs only developed low to moderate resistance to pymetrozine. We also found that pymetrozine can inhibit the fecundity of *N. lugens.*

Establishing a standard bioassay method can help us obtain the relationship between the insecticide dose and pest mortality, thus contributing to our understanding of the evolution of insecticide resistance and helping formulate strategies to delay the development of resistance [20]. The mortality rate is not the only criterion in the insecticide bioassay. In this study, the fecundity assay method was used to calculate insecticide toxicity according to the offspring inhibition rate. For certain kinds of carbamates, benzoylureas, and pyrethroids, people calculate toxicity values according to egg-hatching inhibition rates to determine their ovicidal activity against pests such as *Plutella xylostella* (L.) and *Spodoptera frugiperda* [21,22]. However, an effective resistance monitoring method for pymetrozine needs to assess both its systemic and contact activities. The rice-stem-dipping bioassay method can reflect the two modes of action described above for pymetrozine, and the third-instar nymphs used in the test also meet the requirements concerning field prevention of lower-instar nymphs. The monitoring results of the rice-stem-dipping method showed that pymetrozine was less active against the contemporary *N. lugens* and that *N. lugens* had reached high levels of resistance to pymetrozine (194.6- to 212.8-fold). However, the results of this method did not fully reflect the field efficacy because of the long persistence of pymetrozine and the short duration of the treatment when rice-stem-dipping. The rice-seedling-dipping fecundity assay method examined both the systemic and contact effects of pymetrozine, taking into account its insecticidal mode and mechanism design. Using this method, we found that *N. lugens* only developed moderate resistance to pymetrozine (11.2- to 12.4-fold). In this method, pymetrozine’s toxicity to *N. lugens* was determined by calculating the inhibition rate after different pymetrozine doses from the number of *N. lugens* offspring nymphs. Therefore, this method simultaneously evaluates pymetrozine toxicity in *N. lugens* from the newly hatched nymphs to the third-instar nymphs. The topical application fecundity assay method can only examine the contact effects of pymetrozine. On the basis of this method, we found that *N. lugens* developed low to moderate resistance to pymetrozine (5.4- to 10.8-fold). Both fecundity assay bioassay methods demonstrated that pymetrozine had a significant inhibitory effect on brown planthopper fecundity, and the resistance level monitored by the two methods was basically consistent at low to moderate. This is consistent with the fact that, although the short-term efficacy of pymetrozine against BPHs is decreasing in the current generation, it still has a good field control effect on the next generation due to its long persistence. The fecundity assay method was improved based on the no.005 method released by the IRAC and the topical application method reported by Tsujimoto et al. [17]. This allowed for a more reasonable evaluation of pymetrozine toxicity in BPHs while also being subject to some constraints. As a result of the use of single-pair mating, the fecundity assay method had a large number of replications per concentration and required a large amount of work, which has certain limitations in terms of carrying large-scale resistance monitoring. Combined with the migration habits of *N. lugens*, it is recommended to select three to five field populations of *N. lugens* each year to monitor pymetrozine resistance using this method so as to accurately grasp the resistance dynamics of *N. lugens* to pymetrozine.

In order to determine pymetrozine toxicity in BPHs using the fecundity assay method, this study comprehensively evaluated the effects of pymetrozine’s systemic and contact activity on fecundity in *N. lugens* through rice-seedling-dipping treatment and topical application. As can be seen in our findings, the number of female individuals without offspring increased after pymetrozine treatment, and pymetrozine was shown to inhibit the mating of female and male brown planthoppers. Furthermore, pymetrozine can significantly inhibit the fecundity of *N. lugens*. Wang et al. have confirmed that pymetrozine can activate the TRPV channels, i.e., the *nan* and *iav* complexes, of *N. lugens* and inhibit the reproductive behavior of *N. lugens* and *Drosophila melanogaster* [14,23]. However, the specific effects of pymetrozine on the reproductive processes of female and male *N. lugens* and the physiological, biochemical, and molecular mechanisms through which pymetrozine inhibits male courtship, female receptivity, and oviposition remain to be further studied.

In conclusion, pymetrozine can effectively inhibit the next-generation *N. lugens* population. Combined *N. lugens* control emphasizes the strategy of “suppressing the early population and controlling the late population”, therefore pymetrozine is still recommended for the control of migrating-generation *N. lugens* in order to control the offspring population. Nevertheless, the inhibitory effect of pymetrozine on the fecundity of *N. lugens* requires further attention. Once strong resistance is observed, the fecundity of *N. lugens* cannot be significantly inhibited in this manner. In these cases, the use of pymetrozine should be abandoned and other types of insecticides should be used.

## 5. Conclusions

In summary, we established two fecundity assay bioassay methods to determine the sensitivity of the *N. lugens* pymetrozine-resistant strain and field populations to pymetrozine. Our findings demonstrate that all *N. lugens* strains tested using the traditional rice-stem-dipping method were highly resistant to pymetrozine. The results of the fecundity assay methods showed that pymetrozine could also effectively inhibit the next-generation population of brown planthoppers, and the resistance was low to moderate. Further studies revealed that pymetrozine interferes with the courtship behavior of males and the receptivity behavior and oviposition behavior of females, thus significantly inhibiting the fecundity of *N. lugens*.

## Figures and Tables

**Table 1:** Pymetrozine resistance monitoring of *N. lugens* third-instar nymphs using the rice-stem-dipping method.

Strains	Slope ± SE	χ^2^ (df)	*p* Value	LC_15_ (95%F.L.) mg/L	LC_50_ (95%F.L.) mg/L	LC_85_ (95%F.L.) mg /L	RR ^1^
Pym-S	1.290 ± 0.252	0.59 (3)	0.90	0.422 (0.101–0.843)	2.685 (1.610–4.022)	17.073 (9.840–50.096)	-
Pym-R	1.023 ± 0.236	1.20 (3)	0.75	50.647 (8.000–109.991)	522.520 (311.457–999.180)	5390.8 (2168.5–50,752)	194.6
YZ21	1.263 ± 0.244	0.72 (3)	0.87	83.617 (27.819–146.078)	552.962 (374.727–892.761)	3656.7 (1852.5–14,867)	205.9
QS21	1.328 ± 0.251	1.36 (3)	0.71	94.738 (34.384–160.658)	571.315 (390.675–911.574)	3445.3 (1801.1–12,747)	212.8

^1^ RR: resistance ratio; LC_50_ of Pym-R divided by LC_50_ of Pym-S.

**Table 2:** Susceptibility of *N. lugens* to pymetrozine using two different fecundity assay methods.

Populations	Rice-Seedling-Dipping Fecundity Assay Method					Topical Application Fecundity Assay Method				
	Slope ± SE	χ^2^ (df)	*p* Value	EC_50_ (95%F.L.) (mg/L)	RR	Slope ± SE	χ^2^ (df)	*p* Value	ED_50_ (95%F.L.) (ng/Adult)	RR
Pym-S	0.85 ± 0.11	0.43 (3)	0.93	1.155 (0.882–1.513)	1.0	0.50 ± 0.07	0.34 (3)	0.95	0.052 (0.035–0.079)	1.0
Pym-R	0.89 ± 0.21	0.41 (3)	0.94	14.370 (9.474–21.790)	12.4	0.59 ± 0.08	0.26 (2)	0.97	0.560 (0.350–0.890)	10.8
YZ21	1.09 ± 0.12	0.67 (3)	0.88	12.890 (10.830–15.340)	11.2	0.56 ± 0.07	0.44 (3)	0.93	0.280 (0.200–0.400)	5.4
QS21	0.85 ± 0.35	0.17 (3)	0.98	13.700 (6.619–28.370)	11.9	-	-	-	-	-

## Data Availability

Data is contained within the article.
